# The Attraction of Synchrony: A Hip-Hop Dance Study

**DOI:** 10.3389/fpsyg.2020.588935

**Published:** 2020-12-03

**Authors:** Colleen Tang Poy, Matthew H. Woolhouse

**Affiliations:** ^1^Department of Psychology, Neuroscience and Behavior, McMaster University, Hamilton, ON, Canada; ^2^Digital Music Lab, School of the Arts, McMaster University, Hamilton, ON, Canada; ^3^McMaster Institute for Music and the Mind, McMaster University, Hamilton, ON, Canada

**Keywords:** synchronous, asynchronous, dance, hip-hop, pupillometry, attractiveness

## Abstract

This study investigated an evolutionary-adaptive explanation for the cultural ubiquity of choreographed synchronous dance: that it evolved to increase interpersonal aesthetic appreciation and/or attractiveness. In turn, it is assumed that this may have facilitated social bonding and therefore procreation between individuals within larger groups. In this dual-dancer study, individuals performed fast or slow hip-hop choreography to fast-, medium-, or slow-tempo music; when paired laterally, this gave rise to split-screen video stimuli in which there were four basic categories of dancer and music synchrony: (1) synchronous dancers, synchronous music; (2) synchronous dancers, asynchronous music; (3) asynchronous dancers, one dancer synchronous with music; and (4) asynchronous dancers, asynchronous music. Participants’ pupil dilations and aesthetic appreciation of the dancing were recorded for each video, with the expectation that these measures would covary with levels of synchronization. While results were largely consistent with the hypothesis, the findings also indicated that interpersonal aesthetic appreciation was driven by a hierarchy of synchrony between the dancers: stimuli in which only one dancer was synchronous with the music were rated lower than stimuli in which the dancers were asynchronous with each other and with the music; i.e., stimuli in which the dancers were unequal were judged less favorably than those in which the dancers were equal, albeit asynchronously. Stimuli in which all elements were synchronous, dancers and music, were rated highest and, in general, elicited greater pupil dilations.

## Introduction

Dance is integral to current performing arts and cultural industries worldwide, serving as both a source of entertainment and physical and emotional self-expression. However, dance’s origin predates industries dedicated to culture, and the exact nature of dance’s utility in the evolutionary environment that allows it to persist into the present is uncertain (for extensive discussion concerning the cultural ubiquity of dance, see LaMothe, 2015). There are three primary hypotheses for the evolution of human dance: it evolved (1) as a form of communication, (2) to facilitate social bonding, and/or (3) as an external sign of fitness to attract potential mates.

Communication-driven research hypothesizes that dance evolved to communicate information about an individual and/or propagate cultural information within a society nonverbally. For instance, particular biological movements may communicate a person’s social status, political values, or personality (Hanna, 1987; Luck et al., 2012). Anthropologists suggest that dance was also used to communicate cultural stories, serving a role in telling narratives in folklore and expressing religious values. An example is the Tibetan Buddhists’ *cham* dances, performed as moral lessons promoting compassion for all living things, and as an offering to the guardians in which they believe (Mackerras, 2004). This research considers dance as a form of didactic expression, in which the dancer communicates via actions whose meanings are embedded within the social structures and norms of a specific society (Cohen, 1998). Central to this idea is the ability of humans to simulate (either physically or mentally), based on their body schema, the actions of others. As Vittorio Gallese has stated, “Such personal and body-related experiential knowledge enables us to understand the actions performed by others, and to directly decode the emotions and sensations they experience” (Gallese, 2005). This would mean that not only are dancers able to express emotions and motivation through their movements, but also be understood by their audience.

On a more abstract level, the aesthetic meaning of dance derives from an elaborate relationship between semi-predictable choreographed events, and, in this regard, it is closely related to the concept of musical style. As musicologist Leonard B. Meyer wrote in his book, *Emotion and Meaning in Music*, “styles in music are basically complex systems of probability relationships in which the meaning of any term or series of terms depends upon its relationship with all the other terms possible within the style system” (Meyer, 1956; p. 54). In the case of music, probability relationships are communicated through pitch and rhythm – in dance, via physical gestures. The probability-based approach to aesthetic meaning pioneered by Meyer and others in the 1950’s (e.g., Youngblood, 1956) has had far reaching implications within the field of music cognition and underpins many contemporary approaches (e.g., Cheung et al., 2019).

Socially motivated research hypothesizes that dance evolved to promote prosocial behaviors. For instance, while individuals may use dance to convey emotion, dance’s utility may also be for the benefit of both the observer and the actor. Individuals who can accurately decode emotions from body and limb movement can determine the co-actor’s emotions/intentions and appropriately choose how to respond (Sawada et al., 2003). For example, an observer who identifies an individual as angry may be more likely to withdraw than approach the situation (Harmon-Jones, 2003). Additionally, research indicates that the synchronization component of dance involving multiple participants may signal social affiliation. The “silent-disco” paradigm of Woolhouse et al. (2016) revealed enhanced person-perception and incidental memory for others when dancing in tempo compared to when dancing out of tempo. This increase in interpersonal memory may be the result of synchrony promoting mutual eye contact, enhancing facial-recognition processes (Hood et al., 2003; Macrae et al., 2008). Finally, moving synchronously with others in the context of dance requires coordinated joint action, which necessitates cooperation between individuals (Behrends et al., 2012). Thus in sum, this domain of research suggests that through increasing interpersonal memory, accurate emotional processing, cooperation, and synchrony, dance promotes social affiliation and bonding. For further eye movement research pertaining to dance’s narrative potential, see Ponmanadiyil and Woolhouse (2018).

While dance may aid interpersonal communication, other research suggests that it serves to signal physical and psychological fitness, thereby facilitating mate selection and increasing the likelihood of procreation. For instance, the Big Five personality traits (Costa and McCrae, 1992) have been found to manifest themselves as characteristic movements that observers can accurately identify, which influences perceived dance ability (Luck et al., 2012). Despite that fact that men and women each rate dance ability of the opposite sex differently with respect to the dancer’s combination of personality scores, both rate better dancers as having a higher “datability” (women rated men scoring high on Openness, Conscientiousness, and Neuroticism, but low on Extraversion, and Agreeableness as better dancers, while men rated women scoring high on Extraversion, Openness, and Neuroticism, but low on Conscientiousness, and Agreeableness as better dancers).

Historically, evolutionary psychologists posited that perceptual-cognitive mechanisms exist to solve specific adaptive evolutionary problems, increasing one’s fitness (Buss, 1994/2003). One example is the theory of parental investment of Trivers (1972); mechanisms underpinning mate choices made by biological females evolved to select biological males that optimally increase their fitness. Aggressive and dominant behavior indicated that a male possessed “good” genes, and was subsequently more sexually attractive, but not necessarily generally attractive. However, later researchers have argued that human interpersonal attraction is more multifaceted and complex than Trivers’s analysis (Miller, 2000); which is to say, sexual attraction is related to general attraction, as well as a host of prosocial behaviors, including dance (Graziano et al., 1997).

In light of the research above, we conducted our study investigating the possible role of dance synchrony in enhancing interpersonal aesthetic appreciation. In turn, it is conjectured that this may have facilitated social bonding, mate selection, and therefore procreation between individuals within larger groups. A core principle underpinning our approach is that eye movement behaviors, specifically pupillary dilations, can be used as a proxy for the attractiveness of an observed action. These behaviors are now discussed.

## Overview of Eye Movement Analysis

The oculomotor system is integral to visual perception; eye movements allow an individual to attend to and gather detailed information from particular stimuli in their environment. The region with the highest level of visual acuity is the fovea, the center of the visual field, which fixates on a person’s point of interest. Subsequently, fixations are a measure of a target stimulus’ importance to the observer (Rayner, 2009). While, initial fixations are primarily influenced by a stimulus’ salient low-level properties (i.e., highly contrasting colors, intensity, and spatial orientation), cognitive mechanisms guided by scene-related schemas determine fixation targets and durations. Based on these cognitive mechanisms, fixation targets are categorized as either relevant or irrelevant to the present situation (Henderson, 2003). For example, when instructed to create a sandwich, viewers significantly fixated more on the ingredients and tools required to complete the task – presumably based on their prior knowledge – compared to the irrelevant objects (Rothkopf and Pelz, 2004).

While dissociation between fixation and attention can occur, it is believed to be uncommon (Rayner, 2009). Longer fixations are generally interpreted as the individual attending to a target, presumably to collect relevant information (Hayoe and Ballard, 2005). More complex tasks are associated with longer fixations (Rayner, 2009). Longer dwell time – the summation of fixation durations within a region of interest – indicates greater semantic importance of a specific region to an observer (Henderson, 2003).

The pupil is another ocular structure important for gathering information from the environment. Located at the center of the iris, the pupil allows light information to reach the retina and be sent to the brain for processing. The iris dilates or constricts the pupil to increase or decrease, respectively, the amount of light entering the eye in response to changes in informational salience, hormonal levels, luminance, working-memory load, and emotional arousal (Hyönä et al., 1995; Bradley et al., 2008; Einhäuser et al., 2008; Kang et al., 2014). However, informationally salient-related pupil dilations are significantly smaller than luminance-related dilations, which can cause pupil dilations of up to 4 mm or greater (Beatty and Lucero-Wagoner, 2000). This disparity reflects different biological pathways causing pupil dilation; changes in informational stimuli are associated with sympathetic activation of the superior sympathetic ganglion, while changes in luminance are associated with parasympathetic activation of the Edinger-Westphal nucleus (Heller et al., 1990). Therefore, when using pupil dilation as a measure of informational salience, it is crucial that measurements are made under controlled lighting conditions. In the current study, situating subjects within an artificially lit booth enabled the luminance level to be controlled; see Apparatus Section. For foundational work using pupillometry to examine reward processing, see Murphy et al. (2011), Sepeta et al. (2012), and Mathôt (2018).

## Eye Movements in Dance Observation

While some of the literature’s findings on eye movement behavior in the context of stationary stimuli can be generalized to dynamic human behavior, differences in motion perception and object perception must be considered. Although peripheral vision has lower acuity and is not as efficient in collecting information, it is better at detecting and processing motion. However, observers still use foveal vision to gather detailed information from a point of interest by fixating on or near the target (Rayner, 1998). Thus, peripheral vision can be used by the observer quickly to detect a target’s movement, which allows the observer to shift their gaze such that the target stays in or near the center of their visual field.

Regarding the perception of motion, schemas correspond to expectations of general biological motion, even to the point of specific dance genres. Schematic knowledge of dance is acquired through experience; consequently, the differences in schematic knowledge between novices and experts of dance create different eye movement patterns (Stevens et al., 2010). Experts of dance have greater choreographic knowledge, allowing them to abstract salient information from complex movements more efficiently than novices. As a result, experts of dance have shorter fixation durations than novices, indicating greater cognitive efficiency. Experts also primarily attend to a dancer’s head, while novices attend to their head, neck, torso, and arms more equally. However, after repeated exposure to a piece of choreography, Stevens et al. (2010) found that novices’ eye movement behaviors more closely resembled those of experts, suggesting that experience can increase schematic knowledge rapidly and change eye behaviors in a short period of time.

Dance is usually observed within a musical context, meaning that the rhythmic component of music and its effects on perception must also be considered. Movements synchronous with music facilitate the perception of a musical beat as physical motion, while musically asynchronous movements do not (Arrighi et al., 2009). In instances of audio-visual incongruity, an observer’s eye movements parallel this confusion. A study by Woolhouse and Lai (2014) revealed that the dispersion of fixations – SD of fixation positions between participants at incremental points in time – were greater while observing dancers moving asynchronous to the musical beat. Woolhouse and Lai (2014) proposed that this was due to observers attempting to search for parts of the dancer’s body that was synchronous with the music. Since the asynchronous condition lacked predictable, musically coordinated movements, observers’ search patterns varied greatly, resulting in increased SD values. Contrastingly, conditions in which dancers were synchronous with the music yielded the least overall fixation dispersion values. Furthermore, a dual-dancer study conducted by Woolhouse and Lai (2014) found that observers spent a greater proportion of dwell time on synchronous vs. asynchronous dancers, suggesting that there is an aesthetic preference for audio-visual congruity, at least in the context of dance. For further recent research concerning the relationship between synchrony and aesthetics in the domain of dance, see Vicary et al. (2017) and Howlin et al. (2020).

## Study Objectives

Although dance’s role in enhancing aesthetic appreciation, social bonding, and ultimately facilitating mate selection, is consistent with evolutionary biology and psychology research, the supporting literature arguably lacks empirical breadth. As a result, we sought to determine if changes in dance synchrony corresponded not only with changes in pupil dilation, but also with perceived levels of aesthetic appreciation, i.e., the attractiveness of the dancing using pupillometry and subjective observer ratings. Specifically, the present study investigated whether measurements of aesthetic appreciation positively correlated with dancers’ level of audio-visual synchrony, and thus might provide evidence consistent with the hypothesis that synchronized dance evolved to increase social bonding. Moreover, the study aimed to test the extent to which pupillary dilation can be used as a proxy for the attractiveness of synchronized dancing. As such, we sought to integrate findings across several domains of knowledge to provide evidence for dance’s role in increasing aesthetic appreciation and, by extension, social bonding. In sum, we attempt to offer a more holistic understanding of the role of dance in human and cultural evolution than is perhaps offered in domain-specific studies investigating a single hypothesis, for example, coalition-signaling (Hagen and Bryant, 2003) or mate selection (Miller, 2000, pp. 4–12).

### Hypotheses

Our main hypothesis was that ratings of perceived attractiveness and pupil dilation would positively correlate with levels of dance synchrony – if true, this would be consistent with the notion that dance evolved to enhance the conditions under which social bonding can occur. This study had two additional supporting hypotheses: (1) observers would have longer gaze times for synchronous dancers than asynchronous dancers – if true, this would support the findings of Woolhouse and Lai (2014) of a preference for audio-visual congruity; and (2) pupil dilation would positively correlate with explicit ratings of attractiveness – if true, this would provide evidence that pupil dilations can be used as an implicit measurement of aesthetic appreciation.

## Materials and Methods

### Participants

Around 45 undergraduate students were recruited from the host institution via an online research participation system, and received one course credit as compensation for participating. Seven participants (three males and four females) were excluded from the study due to factors such as infrared glare and undesired body movement that prevented the eye-tracker from collecting their eye movement data consistently throughout the experiment. Thus, data were analyzed for a total of 38 participants (20 males and 18 females), with a mean age of 19.5 years (*SD* = 1.33). All participants had normal or corrected-to-normal eyesight. Participants refrained from wearing heavy eye-make up (e.g., mascara, eyeliner, or eye shadow) or contact lenses to minimize issues preventing accurate collection of eye-tracking data. All participants provided informed consent prior to the experiment.

### Stimuli

The purpose was to present participants with music-dance stimuli, ranging from complete synchrony, in which two dancers danced synchronously in tempo with the music, to complete asynchrony, in which two dancers danced asynchronously with each other out of tempo with the music. To achieve this, four blocks of video stimuli were created, each comprising seven different videos of unique laterally positioned female/male pairs of dancers; this yielded 28 different videos total. Female/male pairs were used to counterbalance possible sex biases between the female and male participants. Each video was approximately 15 s in duration. To minimize carry-over and/or learning effects with respect to the choreography, each participant was presented with only one block. Each block contained (1) music at three different tempi (fast, slow, and medium; Table 1, Column #1), and (2) four levels of dance-music synchrony (“complete synchrony,” “dancer synchrony,” “dancer asynchrony,” and “complete asynchrony”; Table 1, Column #2). For complete synchrony, both dancers moved synchronously with the music and each other; for dancer synchrony, both dancers moved synchronously with each other, but asynchronously with the music; for dancer asynchrony, only one dancer was synchronous with the music, the other was asynchronous; and for complete asynchrony, both dancers were asynchronous with the music and each other.

**Table 1:** Descriptions of the seven videos participants observed in each block.

	#1	#2	#3
Video number	Music tempo	Level of synchrony	Dancers in sync with each other?
1	Fast	Complete synchrony (Dancers synchronous with music)	Yes
2	Slow		
3	Fast	Dancer synchrony (Dancers asynchronous with music)	
4	Slow		
5	Fast	Dancer asynchrony (Single dancer synchronous with music, other asynchronous)	No
6	Slow		
7	Medium	Complete asynchrony (Dancers asynchronous with music)	

**Figure 1:**
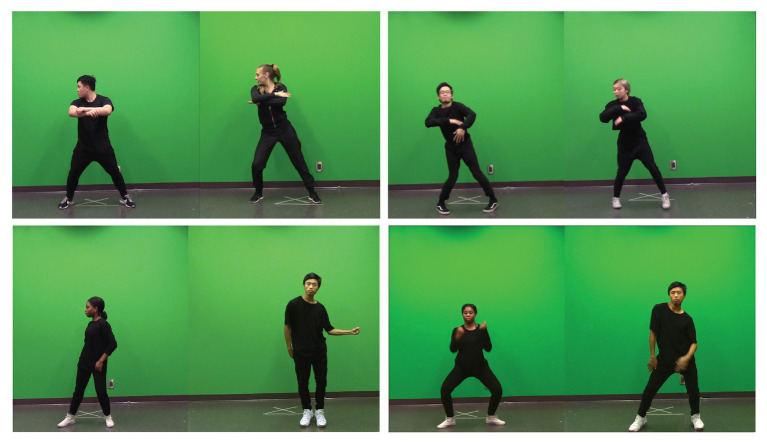
Four video stills showing pairs of dancers dancing synchronously (top, left, and right) and asynchronously (bottom, left, and right).

With respect to the dancers, eight males and eight females specializing in hip-hop dance were filmed using a Canon Vixia HF R600 video camera, recording at 59.89 frames per second. Dancers were between 18 and 26 years old; none of the dancers were members of the institution from which the participants were drawn, thus reducing the risk of a familiarity bias. Each dancer was filmed performing the same two original hip-hop choreographies, the fast- and slow-tempo routines, in front of a monochromatic green-screen wall under the same lighting conditions. These novel routines were choreographed to “So Fine” by Sean Paul [137 beats per minute (bpm)], and “January 28th” by J. Cole (82 bpm), which we categorized as the fast- and slow-tempo music, respectively. Each video was presented with instrumental versions of either “So Fine,” “January 28th,” or the medium-tempo audio track, “No Role Modelz” by J. Cole (100 bpm). These tracks were selected due to their non-whole-integer ratio tempi; unintentional synchrony between the dancers may have resulted had this tempo property not been incorporated into the stimuli. See video stills in Figure 1 for an illustration of pairs of dancers dancing synchronously (top) and asynchronously (bottom).

Participants were instructed to rate the dancing in each video for attractiveness. Participants were informed that the study did not concern sexual attractiveness, but rather the overall aesthetic quality produced by the dancers moving together. As a result, participants’ sexual orientation was not ascertained and did not form part of the analysis. The luminance of the videos was adjusted using Final Cut Pro® to ensure consistent light intensity – i.e., there was neither a perceivable difference in luminance between the left and right dancer within a condition, nor between conditions.

The dancers included in each seven-video block were randomly selected and assigned to each synchrony condition. For the dancer-asynchrony stimuli, the dancer in each pair that performed the choreography associated with the music tempo presented was randomly selected. This ensured that male and female dancers were equally likely to be presented as synchronous with the music in the video stimuli. For the complete-asynchrony stimuli, the choreography each dancer performed was randomly assigned to ensure that both males and females were equally likely to perform either the fast- or slow-tempo choreography. Additionally, the lateral position of each dancer on the screen either left or right, was randomized in order to control for possible visual left-right bias. Finally, the order of the conditions in each block was randomized to minimize order effects.

### Apparatus

Participants’ eye movements were recorded using a bright-pupil Mirametrix S2 tabletop eye-tracker at a sampling rate of 60 Hz. The bright-pupil tracker system – also known as the “red eye effect” caused by on-camera-axis illumination (see Holmqvist et al., 2011, for detailed summary) possesses a 0.5-degree accuracy range, *a* < 0.3 degree drift rating, and allows participants to move their heads within a width-height-depth range of 25 × 11 × 30 cm. This eye-tracker compensates for excessive blinking; the eye-tracker stops recording data and quickly reacquires a participant’s eyes when their eyes move out of sight (i.e., during eye-blinks). The number of eye-blinks per condition did not form part of the analysis.

The Miramex eye-tracker considers a fixation as at least three consecutive points within 40 pixels (Euclidean distance) of each other. The fixation is discarded if there are three invalid frames – where eyes are not detected – or if the next point of gaze is further than the 40 pixel radius. Regarding dwell time, it is calculated from the first of the three points, until the last valid point of the fixation.

Stimuli were presented to participants on a 27″ monitor, with a resolution of 1,920 × 1,080. The participants sat in front of the monitor such the eye-tracking camera, which was set at the base of the monitor, was approximately 2 ft. from the participants’ faces. To minimize on-screen glare and participant distraction, and maintain constant lumination, the experiment was conducted in an artificially lit booth. Participants’ eye-tracking data were exported via EyeMetrix Software. Music was presented through AKG K 172 HD headphones, and set to a comfortable volume by each participant prior to eye-tracking calibration, described below.

### Procedure

Prior to the experiment, the eye-tracker was calibrated for each participant using a nine-point grid, 15-s calibration procedure. Continuation to the video-watching portion of the experiment was only granted if the participant’s calibration score was less than 40, indicating accurate calibration. Participants were randomly assigned to one of the four possible video blocks. To ensure attendance to the musical beat, participants were asked to tap with the index finger of their dominant hand, while watching the videos. This simple tapping task was chosen because it is not cognitively demanding; people have a natural tendency to seek out and automatically move synchronously with a beat (Sebanz et al., 2006). Completion, rather than performance, on this tapping task was recorded. Following each video, via an on-screen prompt, participants were instructed to rate the attractiveness of the dancing on a seven-point Likert scale: 1 = very unattractive; 7 = very attractive. After confirming their rating response, a 3-s fade to black occurred prior to the next video. The duration of this part of the experiment was approximately 10 min per participant.

Following the video-observation portion of the experiment, participants completed a questionnaire to determine if there was a familiarity bias using a 10-point Likert scale: 1 = dancer was unknown to participant; 10 = dancer was well known to participant.

### Analysis

All statistics were calculated in relation to the 38 participants within the study. Data recorded from the right eye were used in the analysis. The screen was divided into two Regions of Interest (ROI): the left and right hand side of the screen covering either dancer. ROIs were equal in size and only one dancer occupied a single ROI throughout each video. Pupil dilation was calculated by determining the difference in average pupil diameter before and during each video stimulus.

A paired samples *t*-test using dwell times investigated the proportion of dwell time each participant spent observing the left vs. the right ROI; we refer to this independent variable (IV) as *Lateral Bias*.

Using pupil dilations and subjective ratings, one-way within-subjects ANOVAs investigated the influence of two IVs on possible levels of attractiveness, music tempo, and synchronous movement, referred to above in the section entitled *Stimuli*. A two-way within-subject ANOVA using *Tempo* and *Synchrony* was not possible since each *Synchrony* level was not represented at each *Tempo* level. For the music-tempo IV, referred to as *Tempo*, there were three conditions; values per participant per condition were calculated as the mean pupil dilation during videos presented with the same music tempo (Table 1, Column #1). For the synchronous-movement IV, referred to as *Synchrony*, there were four conditions. Similarly to *Tempo*, values per participant per condition were calculated as the mean pupil dilation during videos presented with the same level of synchrony (Table 1, Column #2). In a subsequent analysis, *Synchrony* was collapsed to examine whether differences in pupil dilation and attraction ratings were driven by general dancer synchrony vs. asynchrony, i.e., all synchronous vs. all asynchronous dance conditions (Table 1, Column #3).

Lastly, a Pearson product-moment correlation coefficient was calculated between average normalized attractiveness ratings and pupil dilation to determine if there was an association between the two, i.e., whether pupil dilation was a proxy for the attractiveness of the dancing.

## Results

The mean dancer-familiarity rating was 1.19 (*SD* = 0.92), implying that all the dancers were virtually unknown to the participants. This reflected the fact, as reported earlier, that the dancers and participants were from different institutions. As a result, familiarity ratings played no further part in the analysis.

### Lateral Bias

**Figure 2:**
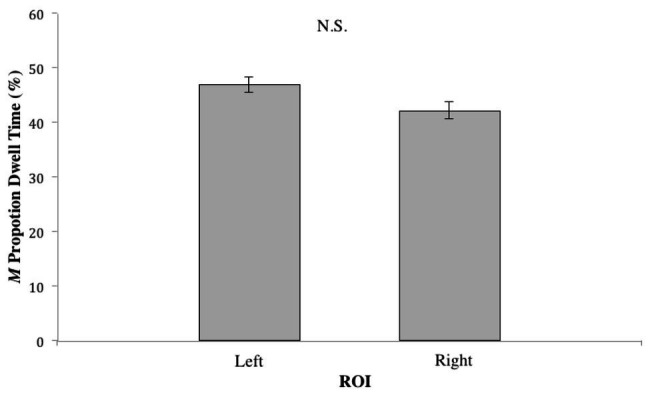
Proportion of dwell time (expressed as %) for the left vs. right Regions of Interests (ROIs) for conditions in which the dancers are synchronous with each other.

**Figure 3:**
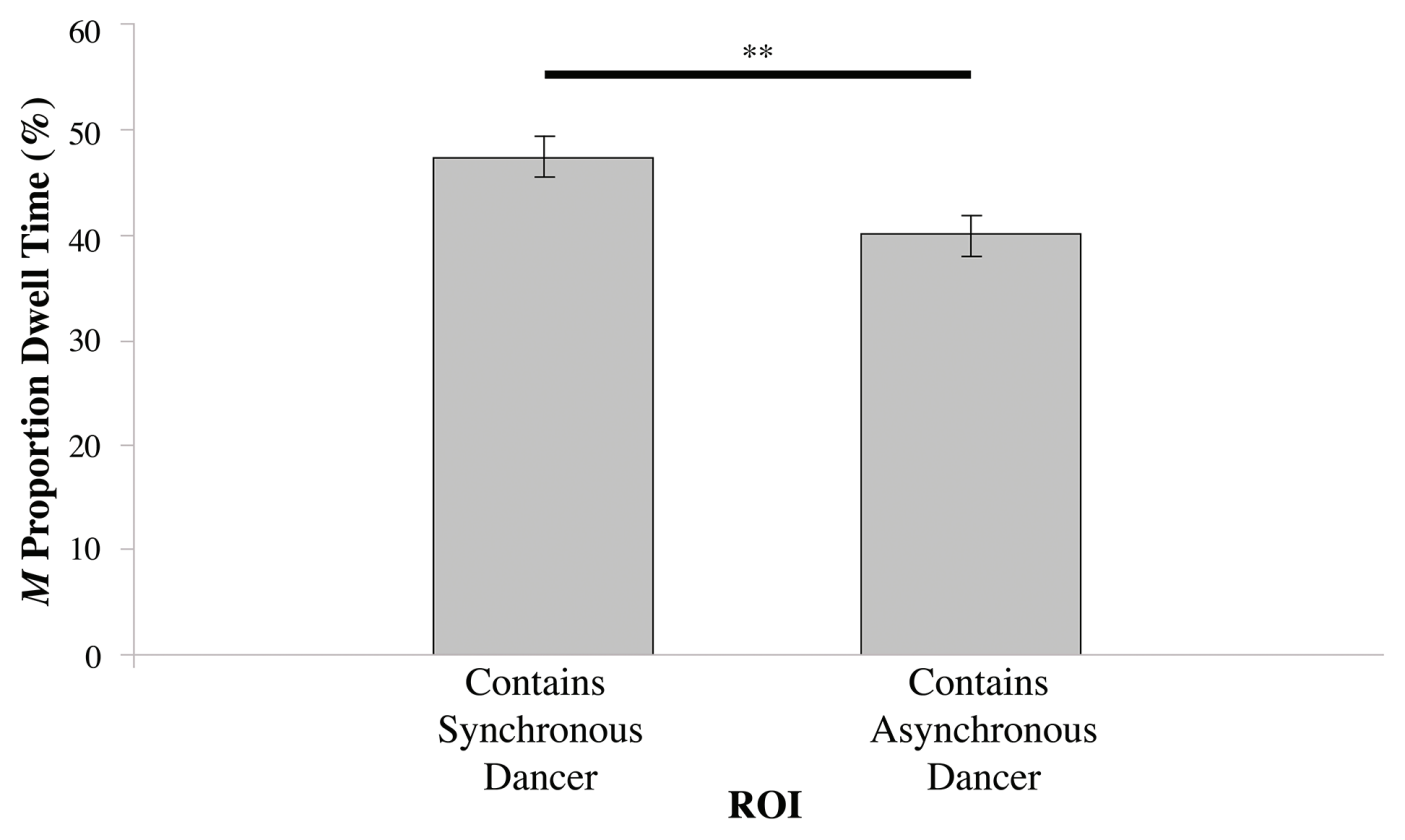
Proportion of dwell time (expressed as %) for the ROI containing the dancer synchronous with the music, vs. the ROI containing the dancer asynchronous with the music in the dancer asynchronous condition (^**^p ≤ 0.01).

For conditions where the dancers were synchronous with each other – Table 1, Videos 1–4 – there was no significant difference in the proportion of dwell time between the left and right ROIs (*t*_75_ = 1.804, N.S.); see Figure 2. However, in the dancer-asynchrony conditions – Table 1, Videos 5–7 – there was a significant difference in the proportion of dwell time between the synchronous ROI vs. asynchronous (*t*_75_ = 2.093, *p* < 0.05). On average, participants spent a greater proportion of dwell time gazing at the synchronous dancer [mean (*M*) = 47.44%, *SD* = 17.29] compared to the asynchronous dancer (*M* = 38.95%, *SD* = 16.06); see Figure 3.

### Synchrony

#### Pupil Dilation

**Figure 4:**
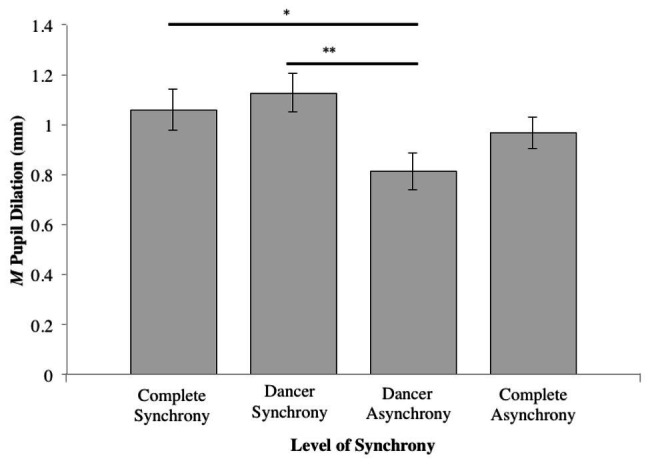
Mean pupil dilation for each level of synchrony (^*^p ≤ 0.05; ^**^p ≤ 0.01).

A significant difference in pupil dilation was found between the *Synchrony* conditions (*F*_3,111_ = 4.16, *η*^2^ = 0.064, *p* < 0.01). Compared to the dancer-asynchrony condition (Table 1, Condition 3; *M* = 0.82, *SD* = 0.08), *post hoc* pairwise comparisons (Tukey HSD) indicated that pupil dilation for dancer-synchrony (Table 1, Condition 2; *M* = 1.13, *SD* = 0.06) was significantly larger (*z* = −0.314, *p* < 0.05). In addition, there was marginally significant larger pupil dilation for the complete-synchrony (Table 1, Condition 1; *M* = 1.06, *SD* = 0.07) compared to dancer-asynchrony (*z* = −0.246, *p* = 0.091). All other pairwise comparisons were not significant (see Table 2 and Figure 4).

**Table 2:** Results of all possible Tukey HSD pairwise comparisons of pupil dilation between *Synchrony* conditions.

Pairs of conditions compared	*z*-value	Significance
Complete synchrony – dancer synchrony	0.068	0.913
Complete synchrony – dancer asynchrony	−0.246	0.091
Complete synchrony – complete asynchrony	−0.091	0.820
Dancer synchrony – dancer asynchrony	−0.314	0.016
Dancer synchrony – complete asynchrony	−0.159	0.424
Dancer asynchrony – complete asynchrony	0.155	0.450

There were 38 data pairs within each pairwise comparison.

**Figure 5:**
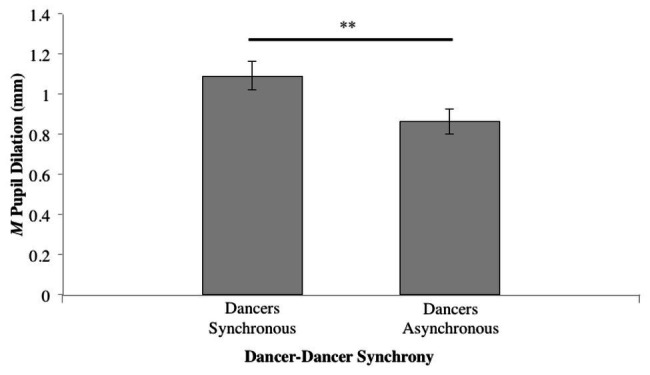
Mean pupil dilation for level of synchrony, while collapsing across both music tempo and dancer-music synchrony (^**^p ≤ 0.01).

When *Synchrony* was collapsed to investigate the general effect of dancer synchrony, a paired samples *t*-test conducted for synchronous (Table 1, Conditions 1 and 2) vs. asynchronous (Table 1, Conditions 3 and 4) revealed a significant difference (*t*_37_ = 2.339, *p* < 0.05); see Figure 5.

#### Attractiveness Ratings

**Figure 6:**
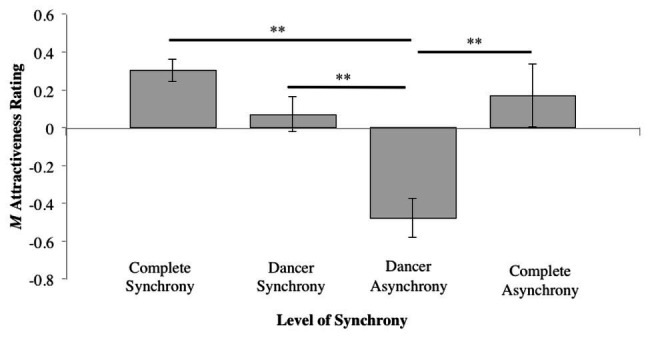
Mean normalized attractiveness rating scores for each level of synchrony (^**^p ≤ 0.01).

A significant difference in attractiveness ratings was found between the *Synchrony* conditions (*F*_3,111_ = 7.67, *η*^2^ = 0.163, *p* < 0.01). Compared to the dancing in the dancer-asynchrony conditions (*M* = −0.48, *SD* = 0.56), *post hoc* pairwise comparisons (Tukey HSD) indicated that dancing in the complete-synchrony (*M* = 0.30, *SD* = 0.35), dancer-synchrony (*M* = 0.07, *SD* = 0.63), and complete-asynchrony conditions (*M* = 0.17, *SD* = 1.02) was rated as significantly more attractive (*z* = −0.236, *p* < 0.01; *z* = −0.236, *p* < 0.01; *z* = −0.650, *p* < 0.01, respectively). All other pairwise comparisons were not significant (see Table 3; Figure 6).

**Table 3:** Results of all possible Tukey HSD pairwise comparisons of subjective attractive ratings between *Synchrony* conditions.

Pairs of conditions compared	*z*-value	Significance
Complete synchrony – dancer synchrony	−0.236	0.439
Complete synchrony – dancer asynchrony	−0.785	0.000
Complete synchrony – complete asynchrony	−0.135	0.827
Dancer synchrony – dancer asynchrony	−0.549	0.004
Dancer synchrony – complete asynchrony	0.101	0.917
Dancer asynchrony – complete asynchrony	0.650	0.000

There were 38 data pairs within each pairwise comparison.

**Figure 7:**
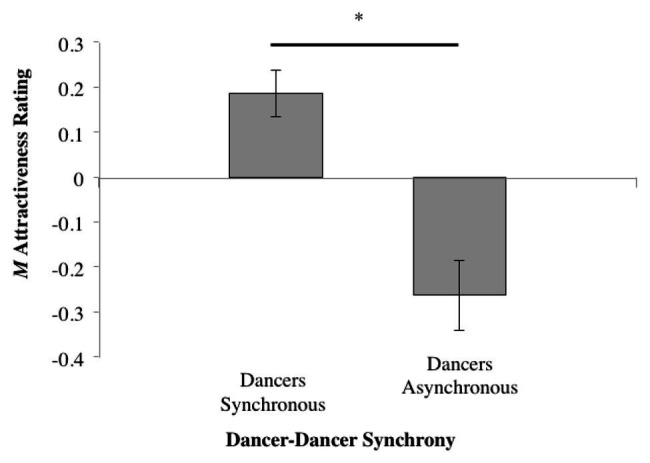
Mean normalized attractiveness rating scores while collapsing across both tempo and dancer-music synchrony (^*^p ≤ 0.05).

Collapsing *Synchrony* to investigate the general effect of dancer synchrony (Table 1, Column #3) revealed that the higher mean rating for the synchronous conditions compared to asynchronous was significant (*t*_37_ = 2.534, *p* = 0.016); see Figure 7.

### Tempo

No effect of *Tempo* was found on pupil dilation (*F*_2,74_ = 0.731, N.S.) or ratings of attractiveness (*F*_2,74_ = 1.121, N.S.). Subsequently, *Tempo* formed no further part of the analysis.

### Attractiveness Ratings and Pupil Dilation

**Figure 8:**
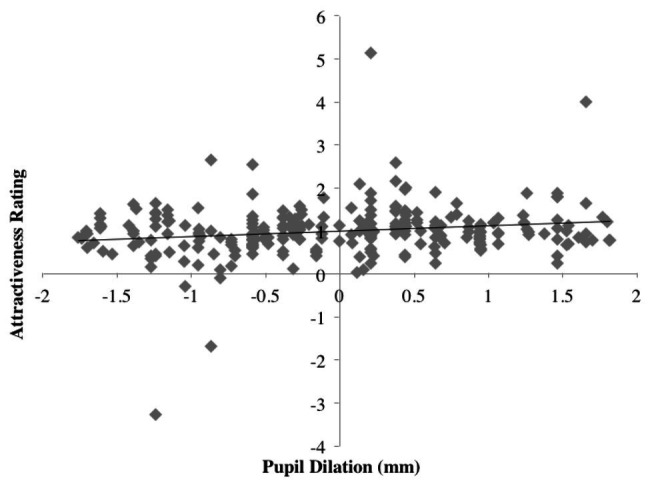
Scatterplot of pupil dilation vs. normalized attractiveness rating score.

A weak positive correlation was found between attractiveness ratings and pupil dilations: *r*(152) = 0.18, *p* < 0.005; two-tailed; see Figure 8.

## Discussion

The overall results of our hip-hop dance study were broadly consistent with the core hypothesis, that synchronized dancing is viewed more favorably than uncoordinated or unsynchronized dancing. This was particularly true of the attractiveness rating data, although the pupil dilation analysis, too, supported this conjecture.

Consistent with the findings of Woolhouse and Lai (2014), in the dancer-asynchrony condition (Table 1, Videos 5–6), participants spent a greater proportion of dwell time observing the ROI containing the dancer moving synchronously with the music, regardless of lateral position. In the synchronous conditions (Videos 1–4), the difference in the proportion of dwell times between the left and right ROIs was not significant. This was expected since neither dancer was more synchronous with the music, thus, participants observed either dancer equally. These findings support the notion of a general preference for audio-visual congruence when observing dance, due, in all likelihood, to greater cognitive efficiency involved in audio-visual integration of compatible visual-aural stimuli (Van der Burg et al., 2010).

A further possible explanation for why we attend to synchronous elements in dance is that we may perceive synchronous multi-person movement as more attractive; the dancing in the synchronous conditions (Videos 1–4) were rated as being more attractive than in asynchronous conditions (Videos 5–7); see Figure 8. Consistent with previous perceptual research, synchronous movement’s greater informational salience was also reflected in increased pupil dilation, while observing synchronous vs. asynchronous conditions (Kang et al., 2014). A larger pupil diameter allows more light information to enter the eye and become available for further processing (see Overview of Eye Movement Behavior Analysis section above). Thus, the greater pupil dilation would allow observers to collect more visual information (from synchronous dancers), allowing them to potentially process and store more interpersonal information, which may in turn enable the creation of elevated levels of affiliation, leading to increased prosocial behaviors.

With respect to the four levels within *Synchrony*, expected and unexpected results were found. As expected, pupil dilation did not significantly differ between conditions in which dancers were synchronous with each other (Videos 1–4), or conditions in which dancers were asynchronous with each other (Videos 5–7). Similarly, there were no differences in attractiveness ratings between the complete-synchrony (Videos 1–2) and dancer-synchrony conditions (Videos 3–4). Furthermore, participants produced greater pupil dilations and gave higher attractiveness ratings to the complete-synchrony and dancer-synchrony conditions than dancer-asynchrony (Videos 5–6), suggesting that the synchronous dancing was perceived to be more attractive than asynchronous dancing.

Unexpectedly, however, the difference in the mean pupil dilation for the complete-synchrony (Videos 1–2) and dancer-asynchrony conditions (Videos 5–6) was only moderately significant. Moreover, mean pupil dilation and ratings for the complete-asynchrony condition (Video 7) were not significantly different from the complete-synchrony (Videos 1–2) and dancer-synchrony conditions (Videos 3–4). These results may be due to the fact that standard pupillometric data analysis, the method adopted here, calculates pupil dilation as the difference between mean pupil diameter prior to and during the video stimulus (Kang et al., 2014). This calculation ignores more fine-grained temporal information that could further expound pupillary dynamics over time. For example, it could be the case that increases in pupil diameter occur only for a short period of time; the current analysis may have allowed early pupil dilations to be masked or dampened by later contractions.

Perhaps most unexpectedly, the attractiveness the dancing in the complete-asynchrony condition (Video 7) was rated as significantly higher than the dancer-asynchrony condition (Videos 5–6). This may be a result of the complete-asynchronous condition appearing comical; observers can perceive skilled individuals behaving incompetently as humorous, and subsequently more appealing and/or attractive (Mettee and Wilkins, 1972, pp. 246–258).

An alternative explanation to the finding reported above, may relate to the fact that perceived attractiveness depends on a *hierarchy* of synchrony between dancers. In the complete-asynchrony condition (Video 7), neither dancer was more synchronous than the other, and, as such, a hierarchy of synchrony was absent. Both the complete-synchrony (Videos 1–2) and dancer-synchrony conditions (Videos 3–4) also lacked a hierarchy of synchrony, which may explain why the attractiveness ratings did not differ significantly from the complete-asynchrony condition’s attractiveness ratings. In contrast, the dancer-asynchrony condition (Videos 5–6) did present a hierarchy of synchrony – only one dancer was synchronous with the music, creating an unequal relationship within the pair. Subsequently, this condition was rated as being significantly less attractive, a finding reinforced by smaller pupil dilations. This suggests that dancing asynchronously to music only negatively affects perceptions of attractiveness if the observer is able to compare the asynchronous dancer to one who is (more) synchronous.

There was no main effect of *Tempo* on pupil dilation or ratings, indicating that the tempo of the music did not affect the measures used in this study to assess either the subjective or objective attractiveness of the dancing; which is to say, the songs used to manipulate *Tempo* – although musically distinct – did not contribute significant variance to the data. Thus, we can infer that any differences in participants’ pupil dilations and attractiveness ratings were more likely due to the manipulation of the other IV, *Synchrony*.

Several limitations to this study may have added uncontrolled-for noise to the data. For example, while dancers were instructed to perform routines as choreographed and with a neutral facial expression, small differences in the velocities of the dancers’ limbs – a result of normal variations in human movement – will undoubtedly have occurred within the stimuli. While these differences did not appear to have a noticeable affect on the synchrony of the videos, and were thus unlikely to have significantly impacted participants’ perceptions, it remains the case that small discrepancies in dancer performance may have influenced participants’ ratings and eye movements. That said, small variations in movement arguably increase the ecological validity of the study, since precise biological movement synchrony within nature is rare due to a host of physiological factors (i.e., age, health, height, weight, sex, etc.).

This study used the medium of hip-hop dance to test our hypotheses. This particular dance style was chosen for two reasons: firstly, choreographically, hip-hop is composed of novel, highly expressive and often angular movements, which strongly align with the musical beat. Stylistically, therefore, it is arguably well suited to a study in which limb and body synchronization must be clearly articulated to observing participants. Secondly, given the relatively youthful demographic of our participants, we were keen to choose a contemporary dance style with which they may have been familiar. That said, similar results may not have been obtained with a different dance style, either because synchronization or coordination between dancers is less obvious, or perhaps less important aesthetically (e.g., as in some contemporary dance). It is therefore an open question regarding the extent to which our results are generalizable.

Furthermore, our experimental design included only seven trials per participant, with two trials representing each level of synchrony except complete asynchrony, which may seem a relatively small number to gather meaningful pupillometry and eye-movement data. In our defense, other eye-tracking and dance studies have also employed a relatively low number of trials, primarily in order to prevent increases in schematic knowledge from exposure to impact eye movement behavior. For example, Stevens et al. (2010) showed participants the same video stimuli as little as twice and found evidence for significant differences in eye movement behavior upon repeated exposure.

With respect to the positive correlation between pupil dilation and attractiveness ratings, any of the explanations for our unexpected results, or the limitations mentioned above, may have weakened the association. In addition, the relatively low coefficient may also have been due to the relatively small sample size, which reduced the statistical power of the correlation. Regardless, despite the circumstances, the resulting significant correlation provides qualified support for the notion that pupil dilation is an implicit measure of the attractiveness of an observed action, in this case synchronized dancing. Future replications of this study could record pupil diameter of participants at a higher temporal resolution, collect data from more participants, and present a larger number of dancers to ameliorate the potentially adverse effects of these limitations.

## Summary

The study’s findings support research showing a preference for audio-visual synchrony in the context of dance, and provide some validation for the use of pupil dilation as a proxy for the attractiveness of synchronized dancing. The results are also consistent with the previously stated hypothesis, that dancers’ level of synchrony affects attractiveness; greater synchrony is perceived as more attractive. This provides evidence for a complementary, alternative hypothesis that the evolutionary utility of synchronized group dancing lies in enhancing the circumstances under which social bonding and affiliation may occur, a hypothesis worthy of further investigation. However, the negative effect of asynchronous dancing on perceived attractiveness is only valid in the context of another synchronous dancer, suggesting that a hierarchy of synchrony, in which one dancer is more synchronous than another, may drive differences in perceived attractiveness.

In conclusion, since dance has been established as an integral part of courtship in other animal species, from Clark’s grebes to peacock spiders (Alcock, 2013), it is possible that a similar evolutionary role of dance exists in humans. While the literature provides evidence that dance promotes prosocial behaviors and facilitates communication, it is conceivable from the evidence of the present study, as well as past research, that dance may also play an additional role in setting the stage for increased interpersonal attraction and thus the conditions under which procreation and group survival may ultimately occur.

## Data Availability Statement

The raw data supporting the conclusions of this article will be made available by the authors, without undue reservation.

## Ethics Statement

The studies involving human participants were reviewed and approved by McMaster Research Ethics Board, McMaster University. The patients/participants provided their written informed consent to participate in this study. Written informed consent was obtained from the individual(s) for the publication of any potentially identifiable images or data included in this article.

## Author Contributions

CT: Study design and execution, data analysis and interpretation, figure and graph creation, and manuscript drafting. MW: Study design and execution, data analysis and interpretation, and manuscript drafting and review. Both the authors contributed to the article and approved the submitted version.

### Conflict of Interest

The authors declare that the research was conducted in the absence of any commercial or financial relationships that could be construed as a potential conflict of interest.
